# Investigating the effects of transcranial direct current stimulation (tDCS) on working memory training in individuals with schizophrenia

**DOI:** 10.1038/s41537-025-00647-5

**Published:** 2025-07-24

**Authors:** Tobias Schwippel, Sanvi Korsapathy, Ibrahim Hajiyev, Aylin Utlu, Simone Weller, Daniel Kamp, Christian Plewnia

**Affiliations:** 1 Department of Psychiatry and Psychotherapy, Neurophysiology & Interventional Neuropsychiatry, University of Tübingen, Tübingen, Germany; 2 Department of Psychiatry, University of North Carolina at Chapel Hill, Chapel Hill, NC USA; 3 Carolina Center for Neurostimulation, University of North Carolina at Chapel Hill, Chapel Hill, NC USA; 4 Department of Psychiatry, Psychotherapy and Psychosomatics, Goethe University Frankfurt, Frankfurt, Germany; 5 DZPG (German Center for Mental Health), partner site Tübingen, Tübingen, Germany; 6 Department of Psychiatry and Psychotherapy, LVR Klinikum Düsseldorf, Medical Faculty, Heinrich-Heine-Universität Düsseldorf, Düsseldorf, Germany

**Keywords:** Schizophrenia, Working memory

## Abstract

Cognitive impairments in schizophrenia significantly impact daily functioning and quality of life, posing a major therapeutic challenge. While both cognitive training and transcranial direct current stimulation (tDCS) have shown promise in improving cognitive function, their combined effects remain underexplored. This double-blind, sham-controlled, randomized clinical trial investigated whether adaptive working memory training (aWMT) paired with anodal tDCS to the right dorsolateral prefrontal cortex (DLPFC) enhances cognitive outcomes in schizophrenia. Twenty-eight individuals with schizophrenia or schizoaffective disorder completed ten sessions of aWMT with concurrent 2 mA anodal or sham tDCS. Cognitive improvements were assessed using the Brief Assessment of Cognition in Schizophrenia (BACS) at baseline, three days after training, and at follow-ups one month, and three months later. Clinical measures evaluated psychopathology, depression, and quality of life. Anodal tDCS significantly improved working memory performance during training, with gains partially sustained at follow-ups. Short-term transfer effects with large effect sizes were observed in the BACS, although clinical symptoms and quality of life remained unaffected. While preliminary, these findings indicate that tDCS could support cognitive training outcomes in schizophrenia. Larger randomized controlled trials are needed to confirm transfer effects and determine the long-term benefits of this approach. If proven effective, integrating tDCS into cognitive remediation therapies could represent an innovative strategy to address cognitive deficits in schizophrenia.

## Introduction

Cognitive impairments are a core symptom of schizophrenia, affecting over 80% of individuals and presenting a significant therapeutic challenge^1,2^. These impairments impact multiple social and neurocognitive domains resulting in substantial difficulties in daily functioning and reducing quality of life^3–5^. Central to these impairments is working memory (WM), which is essential for encoding and retrieving information necessary for goal-directed behavior^6,7^. Neurophysiological studies have linked structural and functional alterations in the dorsolateral prefrontal cortex (DLPFC) to neurocognitive impairments^8,9^. WM deficits specifically, have been associated with reduced grey matter volume, altered functional connectivity from the prefrontal cortex (PFC) to the hippocampus, and reduced excitability of the PFC^8–12^. However, despite evolving neurophysiological understanding, effective, safe and accessible treatments that directly target these neurophysiological alterations have not been established yet.

Cognitive remediation therapy (CRT) is a behavioral training intervention based on scientific principles of learning^13^, proven effective in improving cognitive deficits and increasingly recommended in clinical guidelines^14–17^. A recent meta-analysis demonstrated that CRT improves neurocognition across several domains and global functioning in individuals with schizophrenia^18,19^. However, improvements in global cognition yielded only moderate effect sizes^18–21^. Furthermore, these effects are not consistently maintained during long-term follow-ups and transfer to untrained cognitive domains and global functioning yields only small effect sizes^18,20,22,23^. This is particularly relevant because cognitive training is time- and resource-intensive, often resulting in a disadvantageous cost-benefit ratio^24–27^. Enhancing these effect sizes through augmentation strategies may improve transfer to untrained tasks and reinforce long-term effects, which are critical for optimizing the cost-benefit ratio and promoting broader clinical application.

One possible augmentation strategy is the simultaneous stimulation of brain regions activated during cognitive tasks. For this purpose, transcranial direct current stimulation (tDCS) can be applied concurrently to a cognitive task. By modulating resting membrane potentials in neuronal assemblies beneath the electrode site, tDCS can induce polarity-specific changes in cortical excitability^28^. It is hypothesized that the interplay of endogenous task-dependent activation and exogenous stimulation could result in synergistic effects that amplify the benefits of the training. For WM, increased DLPFC activation which correlated with WM improvement, was observed following working memory training (WMT)^29–31^. Consequently, tDCS was utilized to further enhance DLPFC activity and thus amplify the benefits of WMT. First studies in healthy participants reported that excitatory, anodal tDCS to the DLPFC can effectively enhance WMT, with effects lasting up to 13 months^32^. Furthermore, Ruf and colleagues demonstrated that three days of tDCS-augmented WMT led to improvements in WM, which were maintained at the 9-month follow-up and transferred to an untrained WM task^33^.

In individuals with schizophrenia, single session studies employing anodal tDCS to the DLPFC yielded beneficial effects on WM performance, especially with higher current intensities^34,35^. Studies investigating the effects of recurrent sessions of tDCS without a concurrent cognitive task also demonstrate WM improvements in individuals with schizophrenia^36–39^. Preliminary evidence suggests that combining tDCS with WMT can enhance training gains^40,41^, but further research is needed to determine optimal parameters and establish preliminary efficacy, paving the way for larger RCTs in individuals with schizophrenia.

Our study aims to establish the preliminary efficacy of a 10-day tDCS-augmented WMT for individuals with schizophrenia. Both the task and tDCS parameters were directly informed by our preceding studies^33,34^, and address previous limitations by using a higher tDCS intensity as well as an individualized adaptive WMT (aWMT). We comprehensively assess transfer effects to untrained cognitive domains, clinical symptoms and quality of life, with follow-up assessments up to three months. Compared to sham stimulation, we hypothesized that participants receiving tDCS would show superior WM performance and learning during the aWMT (online effects) and outperform the sham group on WM and transfer to other cognitive domains during follow-up assessments (offline effects), examined at short-term (3 days) and long-term intervals (1 and 3 months).

## Methods

### Study design

**Fig. 1:**
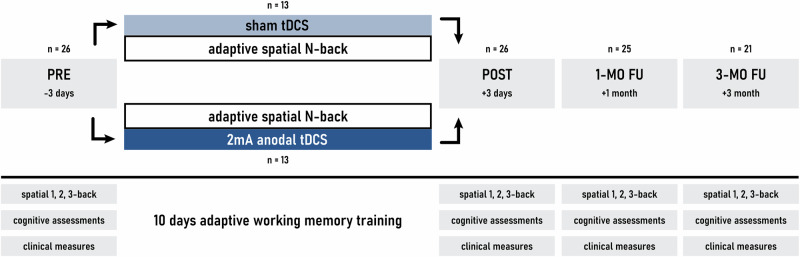
Study design. Cognitive assessments and clinical measures are those listed in Table 1. The training period occurred over the course of 2 weeks (Monday-Friday).

This study was a double-blind, sham-controlled, randomized clinical trial in a parallel design conducted at the Tübingen University Hospital (NCT03621540) and investigated the combination of a 10-day aWMT and anodal tDCS. We explored the effects of the intervention on WM performance, transfer to other cognitive domains, clinical symptomatology, and quality of life with assessments at post-training (3–4 days), the one-month and three-months follow-ups (Fig. 1). This study was approved by the local ethics committees. Written informed consent was obtained from all participants prior to enrollment.

### Participants

Between June 2018 and October 2022, we randomized 37 individuals from the Tübingen and Düsseldorf University Hospitals. Inclusion criteria were diagnosis of schizophrenia or schizoaffective disorder, age 18–60 years, right handedness, stable antipsychotic medication for at least one week before start of the training, sufficient proficiency in German language, and the ability to give informed consent. Exclusion criteria were epilepsy, metallic material in the head area, cardiac pacemaker, significant structural brain abnormalities, prior stroke, prior brain surgery, neurodegenerative disorder, severe somatic comorbidity, acute suicidal ideations, pregnancy, current substance abuse (excluding tobacco), use of anticonvulsive medication, and benzodiazepines equivalent to >1 mg lorazepam. All participants received monetary compensation (150€) for their time and had to remain on stable antipsychotic medication until the post-training session.

Based on previous studies with a comparable design, the initial recruitment target was set at 66 participants. However, due to substantial recruitment difficulties related to the COVID-19 pandemic, only 37 participants could be randomized. Additionally, data from the Düsseldorf study site were excluded due to software malfunctions affecting the computerized task. Of the 28 participants randomized at the Tübingen site, two did not complete the post-training session, resulting in a final per-protocol sample of 26 participants and 344 experimental sessions.

### Transcranial direct current stimulation (tDCS)

The neuroConn DC-Stimulator Plus® (neuroConn GmbH) was used to provide tDCS. A 35 cm² tDCS electrode (rectangular, 7 × 5 cm) was coated with 10/20 conductive electrode paste and placed over the right DLPFC in accordance with the 10–20 EEG system (F4). The cathode was attached to the left deltoid muscle. Stimulation intensity was set to 2 mA, with a ramp-up/down of 15 s and stimulation time of 1500 s (total duration 25.5 min). Impedance was kept below 10 kΩ and the manufacturer’s sham mode was employed. A researcher not involved in the study generated the random allocation sequence using block randomization with a fixed block size of six. Stimulation was initiated 60 s before starting the task. If the participant completed the task before the stimulation ended, they remained seated for the remainder of the stimulation.

### Adaptive spatial N-back training

Computerized adaptive working memory training (aWMT) was administered for approximately 22 min (depending on task difficulty) each working day for two consecutive weeks (10 sessions) concurrent with tDCS. The task was programmed with PsychoPy Version 1.83.04^42^ and participants were asked to monitor a series of stimuli (3 × 3 cm blue square) presented for 0.5 s on the screen. Participants were instructed to respond with a right-handed keyboard press as quickly as possible if a stimulus appeared at the same location as *n* stimuli before; no response was required for non-target stimuli. The interstimulus interval was set to 2.5 s and 25% of the stimuli were targets. Each session consisted of 14 blocks with 20 + *n* stimuli. After each block, accuracy was displayed as feedback. Dependent on accuracy, n-back level was either increased (> 65% accuracy), decreased (≤ 40% accuracy), or maintained (> 40 and ≤ 65% accuracy) for the next block. To foster improvement between sessions, the starting n-back level for each day was set to mean n-back level of the previous day –1. The primary outcome was mean n-back level per session and response time.

### Spatial 1, 2, 3-back

During the pre-training, post-training and follow-up sessions, participants performed a spatial 1-, 2-, and 3-back. The task was delivered in six-minute blocks of increasing difficulty, with 60-second pauses. All three n-back levels comprised 120 + *n* stimuli with 25% of them being targets. The primary outcome was the discriminability index d’ (d-prime)^43^ and response time.

### Clinical and cognitive assessments

During a screening session, demographic information and psychiatric history were collected, diagnosis was confirmed by Structured Clinical Interview for DSM Disorders (SCID-I)^44^, and measures of handedness (Edinburgh Handedness Questionnaire)^45^, nicotine dependence (Fagerstrom)^46^, and premorbid intelligence (MWT-B)^47^ were obtained. During the pre-training session, we administered the Brief Assessment of Cognition in Schizophrenia (BACS)^48^, Trail Making Test-B (TMT-B)^49^, Positive and Negative Syndrome Scale (PANSS)^50^, Calgary Depression Scale for Schizophrenia (CDSS)^51^, Scale for the Assessment of Negative Symptoms (SANS)^52^, Global Assessment of Functioning (GAF)^53^, and Quality of Life (WHOQOL-BREF)^54^, with assessments repeated at post-training and at both follow-ups. The PANSS, SANS, CDSS, and GAF were scored by trained psychiatrists (TS, IH).

### Tolerability

After the final training session participants completed an adverse stimulation effect questionnaire including nine items (tingling around the electrode, tingling in the rest of the head, exhaustion, itching, headache, nausea, warmth, metallic taste, other), rated on a five-point Likert scale (1 = not at all, 5 = extremely). Ratings were averaged across items and participants.

### Statistical analysis

Of the 28 participants enrolled at the Tübingen study site, we analyzed data from the 26 who met the criteria for completing the post-training session and attending at least seven training sessions (per-protocol analysis). The intention-to-treat analysis (*n* = 28) is provided in the supplementary material. Statistical analyses were performed using R (Version 4.4.1)^55^ with the lme4^56^ and afex^57^ packages. The significance level was set to 0.05. Only correct responses were considered for response time analysis. Greenhouse-Geisser correction was applied for violations of sphericity. Residuals were visually assessed using Q-Q plots. All outcome variables revealed acceptable residual distributions, except for response times during training, which exhibited the expected kurtosis (5.56). We retained raw data in the main analysis for clarity of interpretation, with log-transformed results provided in the Supplement. Pairwise post-hoc comparison with Tukey correction for multiple testing was performed with the emmeans package^58^ and estimated marginal means and standard errors are reported. We analyzed the immediate (online) effects of tDCS on WM performance and the after-effects (offline) of tDCS separately. The offline analysis consisted of a short-term analysis of post-training performance assessed three days after the intervention, and a long-term analysis including both post-training and follow-up assessments at 1 and 3 months.

#### Analysis of online effects

We utilized linear mixed models (LMM) to account for missing data and to analyze changes over all sessions as LMMs allow for the inclusion of both fixed and random effects, modeling individual variations. The decision on random effects and interactions was based on model comparison using AIC, likelihood ratio tests, and a priori hypotheses (Supplementary Material). The first random-intercept LMM had n-back level as the dependent variable, with condition and session as independent variables and interaction terms. For response time, we applied a random-intercept LMM with condition, session, and n-back level as independent variables. Condition was treatment-coded, with sham tDCS as the reference. Session and n-back level were treated as continuous variables.

#### Analysis of offline effects

For the spatial 1-, 2-, 3-back, we employed two separate analyses investigating both short-term changes at the post-training session and long-term changes including all sessions. Since baseline differences in d’ were identified between conditions, we used adjusted d’ as the dependent variable for our analysis (normalized by subtracting the pre-training d’). For analysis of post-training d’, we conducted a mixed ANOVA with condition as a between-subjects factor and n-back level (1, 2, 3-back) as a within-subjects factor. For analysis of d’ across all sessions, we conducted repeated measures (RM) ANCOVAs, with session as a covariate. For response time, we conducted a mixed ANOVA with condition as a between-subjects factor and n-back level as a within-subjects factor for the post-training session. For long-term effects, an RM-ANOVA was employed, adding session as a within-subjects factor. Finally, we analyzed cognitive (BACS, TMT) and clinical measures (SANS, PANSS) using mixed ANOVAs for post-training (condition as a between-subjects factor) and RM-ANOVAs with session as a within-subjects factor.

## Results

### Participants

We included 26 right-handed individuals (8 female) diagnosed with schizophrenia or schizoaffective disorder in this study. All participants completed at least seven of ten training sessions (*M* = 9.46, *SD* = 0.81), constituting the per-protocol population used for the main analyses. On average, participants were 37 years old, first diagnosed at 27 years, exhibited a PANSS score of 46, and a BACS composite t-score of 37 before the training. Further demographic and clinical information of study participants can be retrieved from Table 1 and S1.

**Table 1:** Clinical Measures and Cognitive Assessments Over Time.

	Pre-Training		Post Training		One-month Follow-Up		Three-month Follow-Up	
	tDCS (*n* = 13)	sham (*n* = 13)	tDCS (*n* = 13)	sham (*n* = 13)	tDCS (*n* = 13)	sham (*n* = 12)	tDCS (*n* = 12)	sham (*n* = 9)
	*M* (*SD*)	*M* (*SD*)	*M* (*SD*)	*M* (*SD*)	*M* (*SD*)	*M* (*SD*)	*M* (*SD*)	*M* (*SD*)
Cognitive Assessments								
**BACS Composite [t]**	40.69 (14.47)	33.23 (13.66)	47.69 (12.89)	36.38 (15.80)	46.92 (11.50)	38.67 (15.38)	46.00 (14.48)	39.33 (16.89)
** Verbal Memory**	50.46 (16.86)	41.92 (14.53)	49.92 (13.32)	46.69 (17.28)	53.00 (14.18)	45.42 (14.87)	55.5 (14.75)	48.22 (16.98)
** Digit Sequencing**	42.31 (11.83)	36.62 (11.04)	47.08 (10.04)	35.31 (12.51)	42.31 (9.78)	35.17 (15.50)	45.33 (11.77)	37.44 (11.26)
** Token Motor Task**	42.31 (18.17)	43.92 (11.09)	49.08 (16.11)	46.00 (11.76)	51.77 (11.13)	49.58 (8.08)	47.17 (18.61)	47.89 (14.08)
** Verbal Fluency**	46.00 (10.38)	39.46 (11.20)	48.38 (12.55)	42.00 (12.84)	46.38 (9.55)	41.75 (10.57)	45.50 (8.48)	42.89 (11.67)
** Symbol Coding**	37.31 (6.77)	31.92 (8.34)	43.38 (6.91)	36.15 (10.42)	42.23 (8.59)	40.92 (13.45)	41.42 (9.39)	40.22 (10.47)
** Tower of London**	47.08 (8.44)	47.69 (10.93)	52.92 (7.63)	45.92 (10.93)	51.92 (9.35)	47.33 (14.47)	50.08 (7.39)	46.33 (8.38)
**TMT-B [norm]**	92.85 (12.78)	84.23 (19.31)	93.00 (16.48)	89.46 (22.43)	94.15 (15.98)	91.73 (22.37)	97.92 (12.59)	93.44 (13.39)
Clinical Measures								
**WHOQOL-BREF**	89.67 (9.55)*	88.23 (16.26)	85.58 (15.25)*	87.08 (16.10)*	90.08 (12.86)	83.42 (15.78)	87.09 (13.19)	84.44 (12.17)
**CDSS**	2.62 (1.98)	4.38 (4.48)	3.00 (3.06)	1.54 (2.57)	2.46 (2.76)	2.83 (4.45)	3.67 (4.23)	4.78 (4.92)
**GAF**	56.23 (11.82)	53.15 (14.4)	58.85 (11.15)	56.00 (15.79)	60.23 (12.72)	53.92 (15.7)	52.58 (16.75)	51.11 (13.92)
**PANSS Total**	43.62 (8.25)	48.08 (12.11)	42.85 (6.03)	44.62 (10.96)	40.54 (7.08)	43.42 (13.65)	44.75 (9.29)	45.33 (9.11)
** Positive**	10.23 (3.61)	9.77 (2.83)	9.69 (2.46)	9.23 (3.42)	9.15 (2.41)	9.17 (2.76)	10.17 (2.04)	9.89 (3.14)
** Negative**	11.69 (3.28)	14.54 (6.13)	11.85 (3.36)	13.54 (5.84)	11.54 (3.82)	13.25 (6.58)	11.83 (5.47)	12.78 (4.41)
** Psychopathology**	23.23 (4.57)	23.77 (5.21)	22.08 (3.75)	21.92 (4.07)	20.46 (3.48)	21.83 (4.15)	22.75 (4.33)	23.78 (4.68)
**SANS Total**	19.08 (12.46)	20.38 (13.68)	20.00 (12.39)	18.31 (13.05)	15.38 (10.86)	22.58 (16.69)	18.17 (15.52)	23.11 (12.83)
** Affective Flattening**	7.15 (6.49)	4.77 (6.83)	8.08 (5.88)	4.77 (6.15)	6.31 (6.32)	8.83 (7.53)	6.25 (6.47)	8.22 (7.61)
** Alogia**	4.31 (4.05)	3.69 (4.17)	4.62 (3.52)	2.54 (3.45)	3.00 (3.70)	3.33 (3.75)	3.42 (3.99)	2.78 (2.44)
** Apathy**	2.38 (2.57)	3.54 (3.23)	2.46 (2.99)	3.31 (2.98)	2.38 (2.6)	2.67 (2.74)	2.42 (3.42)	3.11 (3.06)
** Anhedonia**	4.23 (3.94)	8.23 (6.03)	3.92 (4.01)	6.85 (6.31)	3.23 (3.61)	7.33 (6.92)	5.50 (6.67)	8.00 (6.00)
** Attention**	1.08 (1.55)	1.31 (2.06)	0.92 (2.25)	0.85 (1.77)	0.50 (1.73)	0.50 (1.24)	0.58 (1.73)	0.78 (1.56)

*Pre-Training:* Three days before the first tDCS-augmented training session, *Post-Training:* Three days after the last tDCS-augmented training session, *BACS:* Brief Assessment of Cognition in Schizophrenia, *TMT-B:* Trail-Making Test, *Version B,* *WHOQOL-BREF*: World Health Organization Quality of Life Assessment, *CDSS:* Calgary Depression Scale for Schizophrenia, *GAF:* Global Assessment of Functioning Scale, *PANSS:* Positive and Negative Syndrome Scale, *SANS:* Scale for the Assessment of Negative Symptoms, *M:* mean, *SD:* standard deviation. *n = 12.

### Online effects: adaptive spatial N-back training with concurrent tDCS

#### N-Back Level

**Fig. 2:**
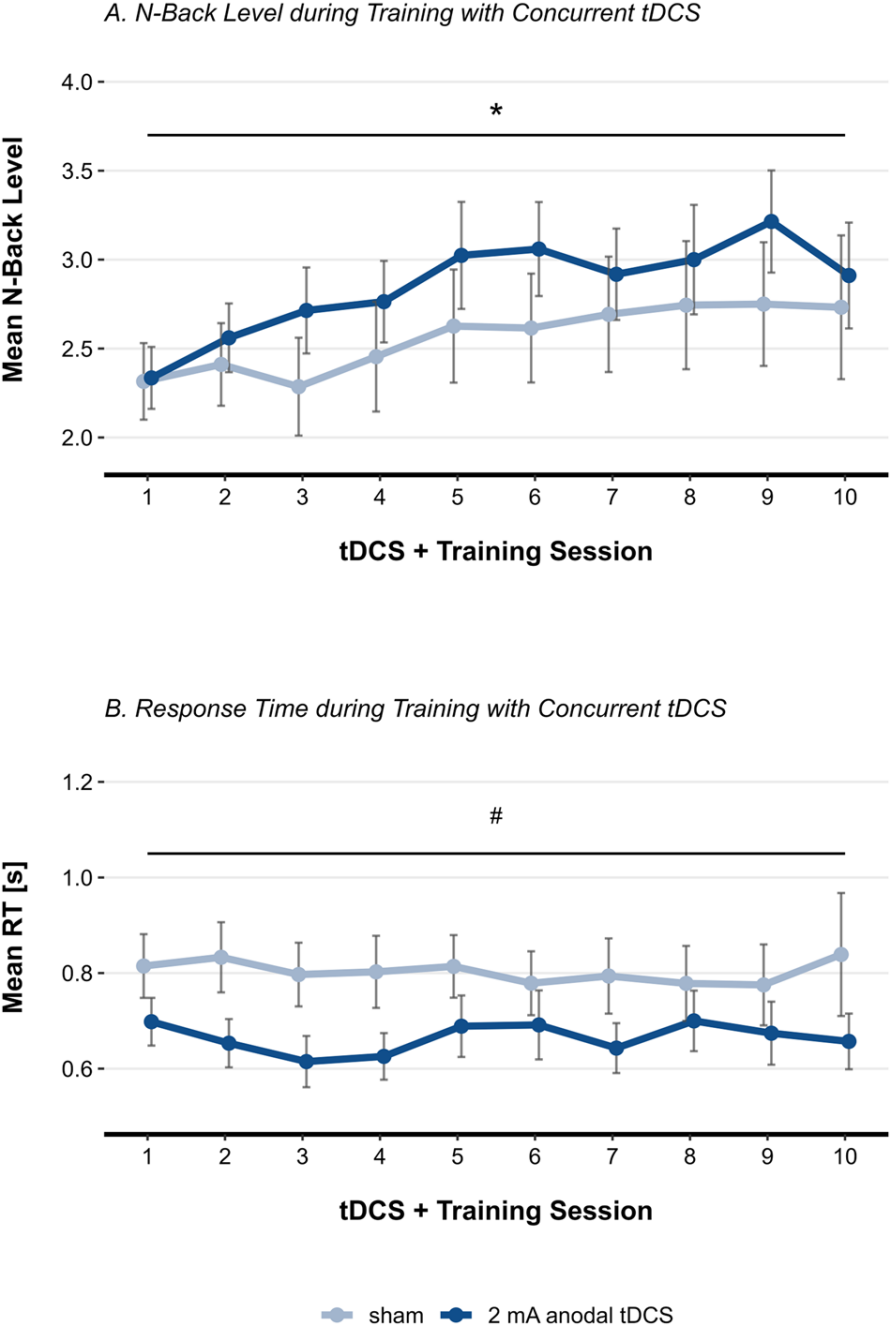
Group Differences in Adaptive Spatial N-Back Training with Concurrent anodal tDCS. **A** Group means of participant-level average n-back level for each training day. The *p*-value represents the interaction effect of condition and session. Error bars represent standard errors. **B** Group means of participant-level average response times for each training day. The *p*-value represents the main effect of condition. Error bars represent standard errors. *p < 0.050, #*p* ≤ 0.070.

The LMM revealed a significant interaction effect between condition and session on n-back level (*b* = 0.022, *t* = 2.284, *p* = 0.022), indicating that the anodal tDCS group achieved higher n-back levels throughout the adaptive n-back training than the sham group (Fig. 2). The post-hoc test did not reveal significant differences between the conditions. Additionally, the LMM yielded a significant main effect of session (*b* = 0.052, *t* = 7.273, *p* < 0.001), highlighting the general effectiveness of the adaptive spatial n-back training. Post-hoc comparisons revealed that n-back level during training sessions 3–10 were significantly higher than at session 1 (all *p* < 0.020).

#### Response Time

According to the LMM, the main effect of condition did not reach significance (*b* = -0.151, *t* = −1.940, *p* = 0.063). The estimate points to a difference in response time of 151 ms between the groups, underlining the observation that the anodal tDCS group consistently responded faster to correct targets across all training sessions, even while performing at a higher task difficulty (Fig. 2). Additionally, a significant main effect of n-back level on response time was observed (*b* = 0.061, *t* = 15.261, *p* < 0.001), indicating that increased task difficulty resulted in slower response times.

The ITT analysis confirmed the robustness of our findings, with no changes in the significance of effects.

### Offline effects: spatial 1,2,3-back

#### D-prime (d’)

**Fig. 3:**
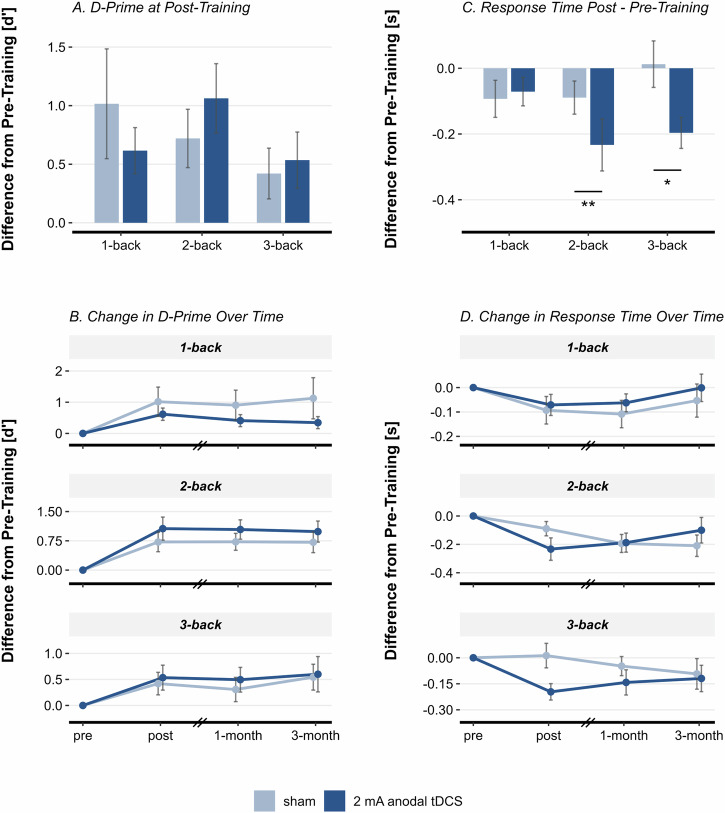
Group Differences in Spatial N-Back Performance Following Stimulation. **A** Mean difference in d’ from pre-training to post-training, split by condition and n-back level. **B** Mean difference in d’ from pre-training across all sessions, split by condition and n-back level. **C** Mean difference in response time from pre-training to post-training, split by condition and n-back level. **D** Mean difference in response time from pre-training across all sessions, split by condition and n-back level. Error bars represent standard error. *p < 0.050, **p < 0.005.

The 2 × 3 ANOVA on d’ post-pre difference score did not yield a significant effect of condition or n-back level. The subsequent RM-ANCOVA including all follow-ups revealed a significant interaction effect of condition and n-back level (*F*(1.82, 123.64) = 4.835, *p* = 0.012, *η*²_*p*_ = 0.066), indicating that anodal tDCS improved d’ compared to sham tDCS depending on n-back level (Fig. 3). Although post-hoc comparisons were not statistically significant, the anodal tDCS group numerically outperformed the sham tDCS group in the 2-back task, with a mean d’ difference of 0.310 points.

#### Response Time

For post-training, the 2 × 3 ANOVA revealed a significant main effect of condition on response time (*F*(1, 23) = 8.369, *p* = 0.008, *η*²_*p*_ = 0.267). Post-hoc comparisons yielded that the anodal tDCS group performed significantly faster than the sham group in the 2-back (*M* = −0.245, *SE* = 0.079, *p* = 0.005), and in the 3-back (*M* = −0.265, *SE* = 0.1123, *p* = 0.027). In the 1-back, the tDCS group did not significantly differ from the sham group (*M* = −0.100, *SE* = 0.052, *p* = 0.068). As anticipated, the ANOVA also yielded a significant main effect of n-back level (*F*(1.5, 34.39) = 5.45, *p* = 0.015, *η*²_*p*_ = 0.192), indicating that response times were slower at higher difficulty levels. Post-hoc comparisons revealed that response times in the 3-back were significantly slower than in the 1-back (*M* = 0.135, *SE* = 0.049, *p* = 0.031).

Next, the RM-ANOVA across all sessions did not reveal a significant main or interaction effect of condition on response times, indicating that the improvement observed in the post-training session with anodal tDCS was not sustained. However, we found a significant main effect of session (*F*(1.77, 31.85) = 8.398, *p* = 0.002, *η*²_*p*_ = 0.318). Post-hoc comparisons revealed that compared to pre-training, response time was significantly lower at post-training (*M* = −0.122, *SE* = 0.029, *p* = 0.003), and one-month follow-up (*M* = −0.135, *SE* = 0.034, *p* = 0.005). We also found a significant effect of n-back level (*F*(1.29, 23.16) = 10.933, *p* = 0.002, *η*²_*p*_ = 0.378). Post-hoc comparisons showed that response times were significantly slower in the 2-back (*M* = 0.077, *SE* = 0.022, *p* = 0.006) and in the 3-back (*M* = 0.144, *SE* = 0.040, *p* = 0.006) compared to the 1-back (Fig. 3). Comprehensive results for d’, response time, hits, false alarms, and criterion c can be obtained from the supplementary material (Tables S4–S6, Figures S1–S2).

### Offline effects: cognitive transfer

#### Brief Assessment of Cognition in Schizophrenia (BACS)

**Fig. 4:**
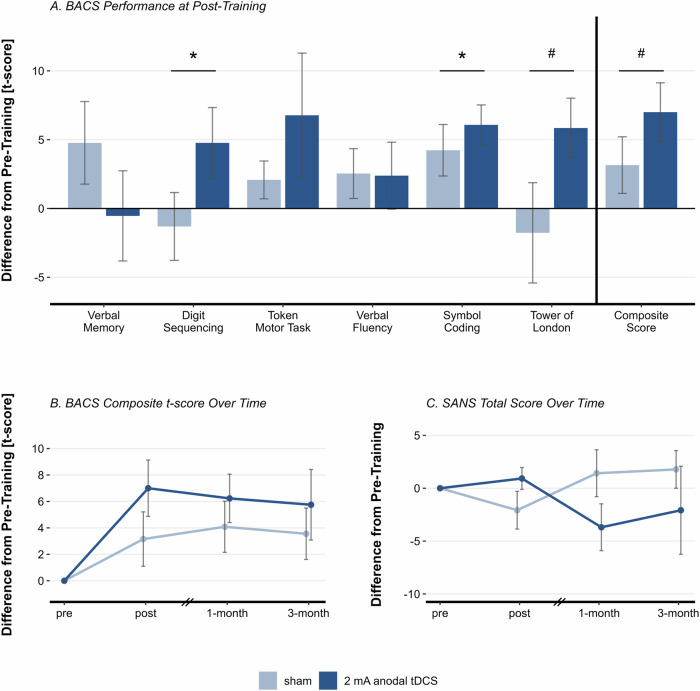
Changes in the Brief Cognitive Assessment for Schizophrenia and Scale for the Assessment of Negative Symptoms. **A** T-score difference from pre-training (pre-training – post-training) for all BACS domains and composite score, split by condition. Error bars represent standard error. **B** Changes in BACS composite t-score over time split by condition. Error bars represent standard error. **C** Changes in SANS total score over time split by condition. Error bars represent standard error. *p < 0.050, #*p* ≤ 0.070.

The ANOVA of post-training BACS composite score did not show a statistically significant main effect of condition (*F*(1, 24) = 3.997, *p* = 0.057, *η*²_*p*_ = 0.143). However, the effect size is large and numerically, the tDCS group improved their t-score by 7 points, whereas the sham group improved by 3.15 points (Fig. 4). Analysis of post-training BACS subscales revealed a significant effect of condition on digit sequencing (*F(*1, 24) = 6.998, *p* = 0.014, *η*²_*p*_ = 0.226) and symbol coding (*F*(1, 24) = 4.346, *p* = .048, *η*²_*p*_ = 0.153). The Tower of London subscale did not reach the significance threshold (*F*(1, 24) = 3.586, *p* = 0.070, *η*²_*p*_ = 0.130), but displayed a medium effect size. All other BACS subscales did not show a significant effect of condition (all *p* > 0.212, all *η*²_*p*_ < 0.065) (Fig. 4).

The RM-ANOVA across all sessions did not yield a significant effect of condition on BACS composite score. However, we found a significant main effect of session with a large effect size (*F*(2.33, 44.18) = 5.326, *p* = .006, *η*²_*p*_ = 0.219) and post-hoc comparisons revealed that the BACS composite score significantly increased from pre-training to post-training (*M* = 5.875, *SE* = 1.41, *p* = .003) and to one-month follow-up (*M* = 5.167, *SE* = 1.422, *p* = 0.009). Albeit numerically still improved compared to pre-training, this effect was statistically not maintained in the three-month follow-up (*M* = 4.653, *SE* = 1.901, *p* = 0.102). With regards to BACS subscales, we did not find a significant interaction of condition and session. A comprehensive summary table of all ANOVAs conducted can be retrieved from the supplementary material (Table S3).

### Offline effects: clinical transfer

#### Scale for the Assessment of Negative Symptoms (SANS)

For total SANS score, neither analysis of post-training nor analysis across all sessions revealed a significant effect of condition or session. Nonetheless, at the one-month follow-up, the tDCS group demonstrated a numerical decrease of 3.7 points, compared to a 2.2 point increase in the sham tDCS group (Fig. 4). With regards to SANS subscales, we did not find a significant effect of condition at post-training, but the RM-ANOVA across all sessions revealed a significant interaction effect of condition and session for affective flattening subscale (*F*(2.79, 53.05) = 3.924, *p* = 0.015, *η*²_*p*_ = 0.171), indicating that anodal tDCS decreased affective flattening. Post-hoc comparisons did not reveal any significant differences between conditions at individual sessions (all *p* > 0.086).

#### Other Clinical Measures

All other clinical measures were analyzed with a post-training ANOVA and RM-ANOVA across all sessions. No significant main or interaction effect for condition was observed for the CDSS, GAF, WHO-QOL-BREF, and PANSS (Table S3). Also, a main effect of session was not observed for any measure, with the exception of the PANSS Psychopathology subscale (*F*(2.13, 40.51) = 4.215, *p* = 0.02, *η*²_*p*_ = 0.182), which decreased throughout the sessions.

### Tolerability and adverse events

No serious adverse events occurred. Overall adverse stimulation effects were rated mild after both tDCS and sham (tDCS: *M* = 1.33, *SD* = 0.60; sham: *M* = 1.31, *SD* = 0.65; Table S2), and did not differ significantly between groups (*t*(11) = −0.118, *p* = 0.908).

### Blinding

Following the last training, participants guessed whether they received sham stimulation. Of the 13 participants who received sham stimulation, five believed they received sham stimulation, and of the 13 that received active tDCS, six believed they received sham stimulation. Fisher’s Exact Test indicated a successful blinding procedure (*p* = 1.000).

## Discussion

This study provides preliminary evidence for an effective augmentation of cognitive training with tDCS in individuals with schizophrenia. Concurrent anodal 2 mA tDCS led to improved WM performance and learning, and effects were partially maintained until follow-ups. Importantly, we report transfer effects to untrained cognitive domains with clinically relevant effect sizes.

### Online tDCS effects on spatial WM

The beneficial effect of concurrent anodal tDCS on WM performance is consistent with findings from our previous studies^33,34^, and is supported by meta-analytic evidence^59^. Both groups showed improved WM capacity throughout the aWMT, though with distinct learning trajectories. tDCS primarily enhanced learning in the early phase (sessions 1–4) before reaching a plateau, whereas the sham group displayed a more gradual learning curve. In contrast, response time did not improve significantly over sessions and did not significantly differ between groups. This is in line with a meta-analysis reporting no effects of online anodal tDCS on response time in neuropsychiatric populations^59^.

The differential effects of tDCS on response time and WM capacity can be attributed to tDCS exerting its influence on two distinct timescales, each governed by different neurophysiological processes^60^. Immediate changes in cortical excitability induced by concurrent tDCS may affect response time, while synaptic plasticity mechanisms, such as long-term potentiation/depression^61,62^, likely contribute to offline consolidation processes between sessions, thereby enhancing WM capacity over time. Recent meta-analyses have also suggested that multiple tDCS sessions may be necessary to produce significant offline effects in individuals with schizophrenia^39,63^, although stimulation was rarely paired with a cognitive task in the investigated studies.

### Offline tDCS effects on spatial WM

At post-training, no significant differences in d’ were observed between the tDCS and sham groups, despite the tDCS group outperforming the sham group during training. The tDCS group showed greater numerical improvements in the 2- and 3-back tasks, while the sham group improved more on the 1-back task, likely due to a ceiling effect, especially within the tDCS group. When including all sessions in the analysis, the significant interaction between condition and task difficulty suggests that tDCS did not uniformly benefit all n-back levels. The 1-back task showed minimal improvement, likely due to its simplicity and the fact that most participants reached maximum performance during training, resulting in a ceiling effect. In contrast, the 2-back task demonstrated the largest gains in d’, consistent with previous findings in individuals with schizophrenia^34,35^. The overall weak offline stimulation effects on d’ might be explained by the fact that the sham group also received the aWMT which likely minimized the observable differences attributable to tDCS.

Although a trend towards faster response time was observed during the concurrent application of tDCS throughout the training sessions, the effects only reached statistical significance in the post-training assessment. Specifically, the tDCS group exhibited significantly faster performance on both the 2-back and 3-back tasks. Here, the non-adaptive n-back task might have allowed the tDCS group to improve the allocation of cognitive resources, previously masked by task switching and within-session learning in the training task. Such optimization potentially resulted in a beneficial speed-accuracy trade-off, allowing participants to respond more rapidly while still preserving their achieved level of stimulus discrimination.

### Offline tDCS transfer to cognition

At post-training, the tDCS group showed more than twice the improvement in the BACS composite t-score compared to the sham group, with better performance in most subscales, except notably for verbal memory and verbal fluency. This specificity might be explained by the laterality of verbal (left) and spatial (right) WM networks^64–66^, leading to laterality-specific improvements in WM depending on tDCS montage^33^. In this study, excitatory tDCS to the right DLPFC might have strengthened the prefrontal part of the visuo-spatial attention network^67,68^ thus facilitating transfer effects in tasks like digit sequencing and symbol coding. However, the specificity of this network activation may have limited broader transfer to domains dependent on different neural networks, potentially explaining both the minimal improvement in tasks relying on verbal WM and the lack of significant changes in clinical outcomes. Nevertheless, the delayed numerical decrease in negative symptoms (SANS) observed at follow-up suggests potential longer-term effects of the tDCS intervention, warranting further investigation. Future research might also optimize stimulation by tailoring parameters to individual neurophysiological signatures and exploring closed-loop approaches to align stimulation with neural activity^69^. Personalized cognitive remediation plans^70^ targeting multiple scales through the training of diverse cognitive domains, specifically enhanced by network-level tDCS augmentation, could further advance far transfer and real-life impact.

### Limitations

Several limitations should be considered in interpreting our findings. First, due to recruitment challenges related to the COVID-19 pandemic, we were unable to reach our planned sample size of 66 participants, which reduced the statistical power, particularly relevant for transfer measures. Second, although participants’ pharmacological treatments were stable until the post-training session, ongoing treatment as usual introduced variability into the data. Third, we observed a ceiling effect in the 1-back task, especially in the tDCS group, which resulted in limited room for further improvement.

## Conclusion

This study contributes to the advancement of cognitive rehabilitation in schizophrenia by providing preliminary evidence that tDCS might enhance the effectiveness of cognitive training. Our findings support the need for a large, multi-center randomized controlled trial to confirm these effects and to more robustly detect transfer effects across cognitive domains and on real-world functioning. The ability of tDCS to enhance the effect size of cognitive training may pave the way for the much-needed broader implementation of CRT in clinical practice.

## Supplementary information

Supplementary Material

CONSORT Flowchart

## Data Availability

Data and code used in this study are available from the corresponding author upon reasonable request. All shared data will be fully anonymized to protect the privacy of individual participants.
