# Supplementary material for: Investigating the effects of transcranial direct current stimulation (tDCS) on working memory training in individuals with schizophrenia
Source: Schizophrenia (Heidelb). 2025 Jul 24;11(1):106. doi: 10.1038/s41537-025-00647-5 (PMC12290004; doi:10.1038/s41537-025-00647-5)
Supplement: Supplementary file 1 — Supplementary Material [file 41537_2025_647_MOESM1_ESM.docx]

**SUPPLEMENTARY MATERIAL**

**Investigating the Effects of Transcranial Direct Current Stimulation (tDCS) on Working Memory Training in Individuals with Schizophrenia**

Tobias Schwippel^a,c,d,e,*^, Sanvi Korsapathy^c,d^, Ibrahim Hajiyev^a,b^, Aylin Utlu^f^, Simone Weller^a,b^, Daniel Kamp^f^, Christian Plewnia^a,b^

a Department of Psychiatry and Psychotherapy, Neurophysiology & Interventional Neuropsychiatry, University of Tübingen, 72076 Tübingen, Germany
b DZPG (German Center for Mental Health), partner site Tübingen

c Department of Psychiatry, University of North Carolina at Chapel Hill, Chapel Hill, NC 27599, USA
d Carolina Center for Neurostimulation, University of North Carolina at Chapel Hill, Chapel Hill, NC 27599, USA
e Department of Psychiatry, Psychotherapy and Psychosomatics, Goethe University Frankfurt, 60528 Frankfurt, Germany
f Department of Psychiatry and Psychotherapy, LVR Klinikum Düsseldorf, Medical Faculty, Heinrich-Heine-Universität Düsseldorf, 40629 Düsseldorf, Germany

***Corresponding Author:** Tobias Schwippel, MD;
Department of Psychiatry, Psychotherapy and Psychosomatics, Goethe University Frankfurt, Heinrich-Hoffmann-Straße 10, 60528 Frankfurt am Main, Germany, schwippel@gmail.com, +4917684090293

**Contents**

**3 Supplementary Methods**

3 Model Derivation: Online Results

3 Intention-to-Treat Sample

**4** **Supplementary Results**

4 Online Effects: Intention-to-Treat (ITT) Analysis

4 Online Effects: Log-Transformed Response Times

5 Table S1. Demographic Information

5 Table S2. Adverse Stimulation Effects

6 Table S3. ANOVA Results of Clinical and Cognitive Measures

7 Table S4. ANOVA Results of Spatial 1,2,3-Back Performance at Post-Training

7 Table S5. RM-ANOVA Results of Spatial 1,2,3-Back Performance Across All Sessions (Main Effects)

8 Table S6. RM-ANOVA Results of Spatial 1,2,3-Back Performance Across All Sessions (Interaction Effects)

9 Figure S1. Group Differences in Hits and False Alarms Following Stimulation

10 Figure S2. Group Differences in Criterion C Following Stimulation

11 Figure S3. Group Differences in Adaptive Spatial N-Back Training for ITT Cohort.

12 Figure S4. Q-Q Plots of Linear Mixed Model Residuals for Response Time during Training.

**Supplementary Methods**

**Linear Mixed Model Derivation: Online Effects**

We used iterative model comparisons to derive the linear mixed model used to analyze training task performance. For both analysis of n-back level and response time, we started with the most basic model including condition as a fixed factor and random intercept of participant. We iteratively added additional parameters based on our priori hypothesis and balanced model complexity with fit using the Akaike Information Criterion (AIC). Factors and interactions were only added to the model and tested when its inclusion aligned with our priori hypothesis. We calculated the difference in AIC (ΔAIC) between models and used likelihood ratio tests to compare models. We repeated this process until the model adequately fit our data and hypothesis while minimizing model complexity.

For analysis of n-back level during training, we conducted a linear mixed model with condition and session as fixed factors and interaction terms, random intercept for participant. Session was coded as a continuous variable, and condition was coded as a factor variable. We used treatment-contrast for condition, with the sham group as the contrast reference.

***N-back level****: n-back level ~ condition * session + (1|ID)*

For analysis of response time during training, we conducted a linear mixed model with condition, session, and n-back level as fixed factors and random intercept for participant. We did not anticipate an improvement (“learning”) of response times over time and the addition of the interaction term session x condition did not improve the model. Session and n-back level were coded as continuous variables, and condition was coded as a factor variable. We used treatment-contrast for condition, with the sham group as the contrast reference.

***Response time:*** *response time ~ condition + session + n-back level + (1|ID)*

**Intention-to-Treat (ITT) Sample**

We conducted an intention-to-treat (ITT) analysis to assess online effects during adaptive training, including all participants who completed at least one training session (n = 28). This extends the per-protocol analysis (n = 26) by two participants. Of these, one participant in the sham group completed a single training session, and one in the tDCS group completed four sessions. Neither completed the post-training assessment. The ITT analysis employed the same statistical model as the per-protocol analysis, and results are reported with bootstrapped 95% confidence intervals.

**Supplementary Results**

**Online Effects: Intention-to-Treat (ITT) Analysis**

N-Back Level. The LMM revealed a significant interaction between condition and session on n-back level (*b* = 0.023, *t* = 2.344, *p* = .019, CI [0.004, 0.042]), which indicates that the anodal tDCS group achieved higher n-back levels over the course of training compared to the sham group (Figure S3). Post-hoc testing did not reveal any significant differences between the two conditions. The LMM also revealed a significant main effect of session (*b* = 0.052, *t* = 7.308, *p* < .001, CI [0.038, 0.066]) and subsequent post-hoc comparisons revealed that n-back level during training sessions 3-10 were significantly higher than at session 1 (all *p* < .020), indicating that regardless of stimulation condition, the training improved performance over time. These results align with our per-protocol analysis.

Response Time. The main effect of condition did not reach significance (b = -0.129, t = -1.605, p = .120, CI [-0.286, 0.035]). The LMM revealed a significant main effect of n-back level on response time (b = 0.063, t = 15.618, p < .001, CI [0.056, 0.070]), indicating that increasing task difficulty slows response time (Figure S3).

**Online Effects: Log-Transformed Response Times**

We plotted the quantile-quantile (Q-Q) distribution of the model residuals for response time during the adaptive spatial n-back and observed a clear deviation from the normal distribution (Figure S4). This was expected as response times are mostly right skewed (skewness = 1.4, kurtosis = 5.6). Thus, we conducted a log transformation on the response time data, which improved this deviation (skewness = 0.5, kurtosis = 3.0) (Figure S4). We then utilized the same LMM on the transformed data in the per-protocol sample. Based on the LMM, the main effect of condition did not reach significance (b = -0.083, t = -1.821, p = .080). The LMM also revealed a significant main effect of n-back level on response time (b = 0.026, t = 12.869, p < .001) indicating that increased task difficulty slowed response time. These results align with our original analysis of the non-transformed data. However, we also found a significant effect of session (b = -0.002, t = -3.586, p < .001), which we did not observe in our original analysis of the non-transformed data. Post hoc comparisons revealed that response time during training sessions 3, 4, 6, 7, and 9 were significantly lower than at session 1 (all p < .047).

| **Table S1.** Demographic Information |  |  |
| --- | --- | --- |
|  | **tDCS** | **sham** |
|  | n = 13 | n = 13 |
| **Age, M (SD)** | 36.92 (12.81) | 38.00 (10.74) |
| **Sex (female)** | 4 | 4 |
| **Education Level** |  |  |
| 9-year high school (Hauptschule) | 2 | 1 |
| 10-year high school (Realschule) | 1 | 2 |
| 13-year high school (Gymnasium/Abitur) | 5 | 2 |
| University Degree | 2 | 3 |
| Vocational Training/Apprenticeship | 3 | 5 |
| **Edinburgh Handedness** | 92.31 (9.24) | 91.31 (10.73) |
| **Smoking Status (yes)** | 5 | 6 |
| **Fagerstrom, M (SD)** | 4.80 (2.95) | 5.83 (2.56) |
| **MWT-B (norm), M (SD)** | 94.00 (10.88) | 89.38 (7.73) |
| **Age of Onset, M (SD)** | 26.77 (9.64) | 28.15 (6.63) |
| **Hospitalizations** | 7.85 (13.22) | 5.54 (6.63) |
| MWT-B: Multiple-Choice Word Test, M: mean, SD: standard deviation. | | |

| **Table S2.** Adverse Stimulation Effects | | |  |
| --- | --- | --- | --- |
| Adverse Effect | **tDCS** | **sham** |  |
|  | *M* (*SD*) | *M* (*SD*) | *p* |
| **Tingling at electrode site** | 2.15 (0.38) | 1.92 (0.79) | .343 |
| **Tingling on rest of head** | 1.00 (0.00) | 1.17 (0.39) | .136 |
| **Exhaustion** | 1.38 (0.51) | 1.67 (1.07) | .403 |
| **Itching** | 1.62 (0.77) | 1.33 (0.65) | .334 |
| **Headache** | 1.46 (0.97) | 1.08 (0.29) | .206 |
| **Nausea** | 1.00 (0.00) | 1.08 (0.29) | .308 |
| **Warmth** | 1.23 (0.44) | 1.17 (0.39) | .704 |
| **Metallic** | 1.00 (0.00) | 1.17 (0.58) | .308 |
| **Other** | 1.09 (0.30) | 1.18 (0.60) | .660 |
| All adverse stimulation effects are rated from 1-5; 1: Not at all; 2: A little; 3: Reasonably; 4: Considerably; 5: Extremely. One participant did not complete the questionnaire. M: mean, SD: standard deviation. | | | |

| **Table S3.** ANOVA Results of Clinical and Cognitive Measures | | | | | | | | | | | | | | | |
| --- | --- | --- | --- | --- | --- | --- | --- | --- | --- | --- | --- | --- | --- | --- | --- |
|  | **ANOVA at Post-Training** | | |  | **RM-ANOVA Across All Sessions** | | | | | | | | | | |
|  | **condition** | | |  | **condition** | | |  | **session** | | |  | **condition x session** | | |
| Cognitive Assessment | ***df*** | ***p*** | ***η²*** |  | ***df*** | ***p*** | ***η²*** |  | ***df*** | ***p*** | ***η²*** |  | ***df*** | ***p*** | ***η²*** |
| **WHOQOL-BREF** | F(1, 22) | .817 | 0.002 |  | F(1, 17) | .956 | 0.000 |  | F(2.21, 37.56) | .451 | 0.047 |  | F(2.21, 37.56) | .679 | 0.024 |
| **BACS Composite** | F(1, 24) | .057 | 0.143 |  | F(1, 19) | .261 | 0.066 |  | **F(3, 57)** | **.003** | **0.219** |  | F(3, 57) | .491 | 0.041 |
| **Verbal Memory** | F(1, 24) | .598 | 0.012 |  | F(1, 19) | .333 | 0.049 |  | F(3, 57) | .340 | 0.057 |  | F(3, 57) | .906 | 0.010 |
| **Digit Sequencing** | **F(1, 24)** | **.014** | **0.226** |  | F(1, 19) | .097 | 0.138 |  | F(3, 57) | .395 | 0.051 |  | F(3, 57) | .151 | 0.088 |
| **Token Motor Task** | F(1, 24) | .583 | 0.013 |  | F(1, 19) | .940 | 0.000 |  | **F(3, 57)** | **.035** | **0.139** |  | F(3, 57) | .553 | 0.036 |
| **Verbal Fluency** | F(1, 24) | .212 | 0.064 |  | F(1, 19) | .414 | 0.035 |  | F(3, 57) | .543 | 0.037 |  | F(3, 57) | .889 | 0.011 |
| **Symbol Coding** | **F(1, 24)** | **.048** | **0.153** |  | F(1, 19) | .527 | 0.021 |  | **F(2.26, 42.88)** | **<.001** | **0.316** |  | F(2.26, 42.88) | .385 | 0.050 |
| **Tower of London** | F(1, 24) | .070 | 0.130 |  | F(1, 19) | .284 | 0.060 |  | F(3, 57) | .440 | 0.046 |  | F(3, 57) | .297 | 0.062 |
| **TMT-B (norm)** | F(1, 24) | .651 | 0.009 |  | F(1, 18) | .666 | 0.011 |  | F(2.2, 39.59) | .150 | 0.098 |  | F(2.2, 39.59) | .577 | 0.032 |
| Clinical Measure |  |  |  |  |  |  |  |  |  |  |  |  |  |  |  |
| **CDSS** | F(1, 24) | .199 | 0.068 |  | F(1, 19) | .418 | 0.035 |  | F(1.9, 36.02) | .121 | 0.106 |  | F(1.9, 36.02) | .082 | 0.125 |
| **GAF** | F(1, 24) | .600 | 0.012 |  | F(1, 19) | .609 | 0.014 |  | F(1.73, 32.86) | .241 | 0.073 |  | F(1.73, 32.86) | .677 | 0.018 |
| **PANSS Total** | F(1, 24) | .615 | 0.011 |  | F(1, 19) | .480 | 0.027 |  | F(2.3, 43.68) | .104 | 0.109 |  | F(2.3, 43.68) | .523 | 0.035 |
| **Positive** | F(1, 24) | .696 | 0.006 |  | F(1, 19) | .894 | 0.001 |  | F(3, 57) | .461 | 0.044 |  | F(3, 57) | .987 | 0.002 |
| **Negative** | F(1, 24) | .374 | 0.033 |  | F(1, 19) | .438 | 0.032 |  | F(3, 57) | .487 | 0.042 |  | F(3, 57) | .555 | 0.036 |
| **Psychopathology** | F(1, 24) | .921 | 0.000 |  | F(1, 19) | .564 | 0.018 |  | **F(2.13, 40.51)** | **.020** | **0.182** |  | F(2.13, 40.51) | .680 | 0.021 |
| **SANS Total** | F(1, 24) | .737 | 0.005 |  | F(1, 19) | .686 | 0.009 |  | F(1.98, 37.69) | .705 | 0.018 |  | F(1.98, 37.69) | .173 | 0.088 |
| **Affective Flat.** | F(1, 24) | .174 | 0.076 |  | F(1, 19) | .623 | 0.013 |  | F(3, 57) | .421 | 0.048 |  | **F(3, 57)** | **.013** | **0.171** |
| **Alogia** | F(1, 24) | .142 | 0.088 |  | F(1, 19) | .409 | 0.036 |  | F(3, 57) | .059 | 0.122 |  | **F(3, 57)** | **.049** | **0.128** |
| **Apathy** | F(1, 24) | .477 | 0.021 |  | F(1, 19) | .135 | 0.114 |  | F(3, 57) | .634 | 0.029 |  | F(3, 57) | .760 | 0.020 |
| **Anhedonia** | F(1, 24) | .171 | 0.076 |  | F(1, 19) | .125 | 0.119 |  | F(2.02, 38.34) | .211 | 0.079 |  | F(2.02, 38.34) | .528 | 0.033 |
| **Attention** | F(1, 24) | .924 | 0.000 |  | F(1, 18) | .932 | 0.000 |  | F(2.27, 40.86) | .313 | 0.063 |  | F(2.27, 40.86) | .983 | 0.001 |
| BACS: Brief Assessment of Cognition in Schizophrenia; TMT-B: Trail-Making Test, Version B; WHOQOL-BREF: World Health Organization Quality of Life Assessment; CDSS: Calgary Depression Scale for Schizophrenia; GAF: Global Assessment of Functioning Scale; PANSS: Positive and Negative Syndrome Scale; SANS: Scale for the Assessment of Negative Symptoms. Significant results highlighted in bold. | | | | | | | | | | | | | | | |

| **Table S4.** ANOVA Results of Spatial 1-,2-,3-Back Performance at Post-Training | | | | | | | | | | | |
| --- | --- | --- | --- | --- | --- | --- | --- | --- | --- | --- | --- |
|  | **condition** | | |  | **nbacklevel** | | |  | **condition x n-back level** | | |
| **Metric** | ***df*** | ***p*** | ***η²*** |  | ***df*** | ***p*** | ***η²*** |  | ***df*** | ***p*** | ***η²*** |
| **d'** | F(1, 23) | .951 | <0.001 |  | F(1.85, 42.52) | .224 | 0.063 |  | F(1.85, 42.52) | .320 | 0.048 |
| **RT** | **F(1, 23)** | **.008** | **0.267** |  | **F(1.5, 34.39)** | **.015** | **0.192** |  | F(1.5, 34.39) | .115 | 0.096 |
| **hits** | F(1, 23) | .156 | 0.085 |  | **F(1.86, 42.68)** | **<.001** | **0.620** |  | F(1.86, 42.68) | .845 | 0.007 |
| **false alarms** | F(1, 23) | .296 | 0.047 |  | **F(1.21, 27.81)** | **<.001** | **0.428** |  | F(1.21, 27.81) | .731 | 0.007 |
| **criterion c** | F(1, 23) | .519 | 0.018 |  | **F(1.97, 45.25)** | **<.001** | **0.387** |  | F(1.97, 45.25) | .762 | 0.012 |
| RT: response time, d’: d-prime. ANOVA formula: dependent variable ~ condition * n-back level. Significant results highlighted in bold. | | | | | | | | | | | |

| **Table S5.** RM-ANOVA Results of Spatial 1-,2-,3-Back Performance Across All Sessions (Main Effects) | | | | | | | | | | | |
| --- | --- | --- | --- | --- | --- | --- | --- | --- | --- | --- | --- |
|  | **condition** | | |  | **session** | | |  | **n-back level** | | |
| **Metric** | ***df*** | ***p*** | ***η²*** |  | ***df*** | ***p*** | ***η²*** |  | ***df*** | ***p*** | ***η²*** |
| **d'** | F(1, 68) | .842 | 0.001 |  | F(1, 68) | .927 | <0.001 |  | F(1.82, 123.64) | .496 | 0.010 |
| **RT** | F(1, 18) | .078 | 0.163 |  | **F(1.77, 31.85)** | **.002** | **0.318** |  | **F(1.29, 23.16)** | **.002** | **0.378** |
| **Hits** | F(1, 18) | .172 | 0.101 |  | **F(1.82, 32.68)** | **< .001** | **0.516** |  | **F(1.91, 34.36)** | **< .001** | **0.818** |
| **false alarms** | F(1, 18) | .227 | 0.080 |  | F(1.16, 20.94) | .484 | 0.031 |  | **F(1.17, 21.09)** | **.004** | **0.346** |
| **criterion c** | F(1, 18) | .634 | 0.013 |  | **F(2.23, 40.14)** | **.004** | **0.246** |  | **F(1.61, 29.03)** | **< .001** | **0.675** |
| \| RT: response time, d’: d-prime. ANOVA formula for d’: d’ difference from pre-training ~ condition * n-back level + session. ANOVA formula for all other dependent variables: dependent variable ~ condition * n-back level * session. Significant results highlighted in bold. \| \| --- \| | | | | | | | | | | | |

| **Table S6.** RM-ANOVA Results of Spatial 1-,2-,3-Back Performance Across All Sessions (Interaction Effects) | | | | | | | | | | | | | | | |
| --- | --- | --- | --- | --- | --- | --- | --- | --- | --- | --- | --- | --- | --- | --- | --- |
|  | **condition x session** | | |  | **condition x n-back level** | | |  | **n-back level x session** | | |  | **condition x n-back level x session** | | |
| **Metric** | ***df*** | ***p*** | ***η²*** |  | ***df*** | ***p*** | ***η²*** |  | ***df*** | ***p*** | ***η²*** |  | ***df*** | ***p*** | ***η²*** |
| **d'** | ---------------- | -------- | --------- |  | **F(1.82, 123.64)** | **.012** | **0.066** |  | F(1.82, 123.64) | .856 | 0.002 |  | ----------------- | -------- | ----------- |
| **RT** | F(1.77, 31.85) | .151 | 0.102 |  | F(1.29, 23.16) | .675 | 0.014 |  | F(3.67, 66.13) | .158 | 0.088 |  | F(3.67, 66.13) | .325 | 0.062 |
| **hits** | F(1.82, 32.68) | .194 | 0.088 |  | F(1.91, 34.36) | .928 | 0.004 |  | F(3.06, 55.09) | .445 | 0.048 |  | F(3.06, 55.09) | .217 | 0.078 |
| **false alarms** | F(1.16, 20.94) | .417 | 0.040 |  | F(1.17, 21.09) | .323 | 0.057 |  | F(2.21, 39.87) | .203 | 0.084 |  | F(2.21, 39.87) | .619 | 0.028 |
| **criterion c** | F(2.23, 40.14) | .290 | 0.067 |  | F(1.61, 29.03) | .833 | 0.007 |  | F(3.51, 63.24) | .484 | 0.045 |  | F(3.51, 63.24) | .300 | 0.065 |
| RT: response time, d’: d-prime. RM-ANOVA formula for d’: d’ difference from pre-training ~ condition * n-back level + session. RM-ANOVA formula for all other dependent variables: dependent variable ~ condition * n-back level * session. | | | | | | | | | | | | | | | |

| 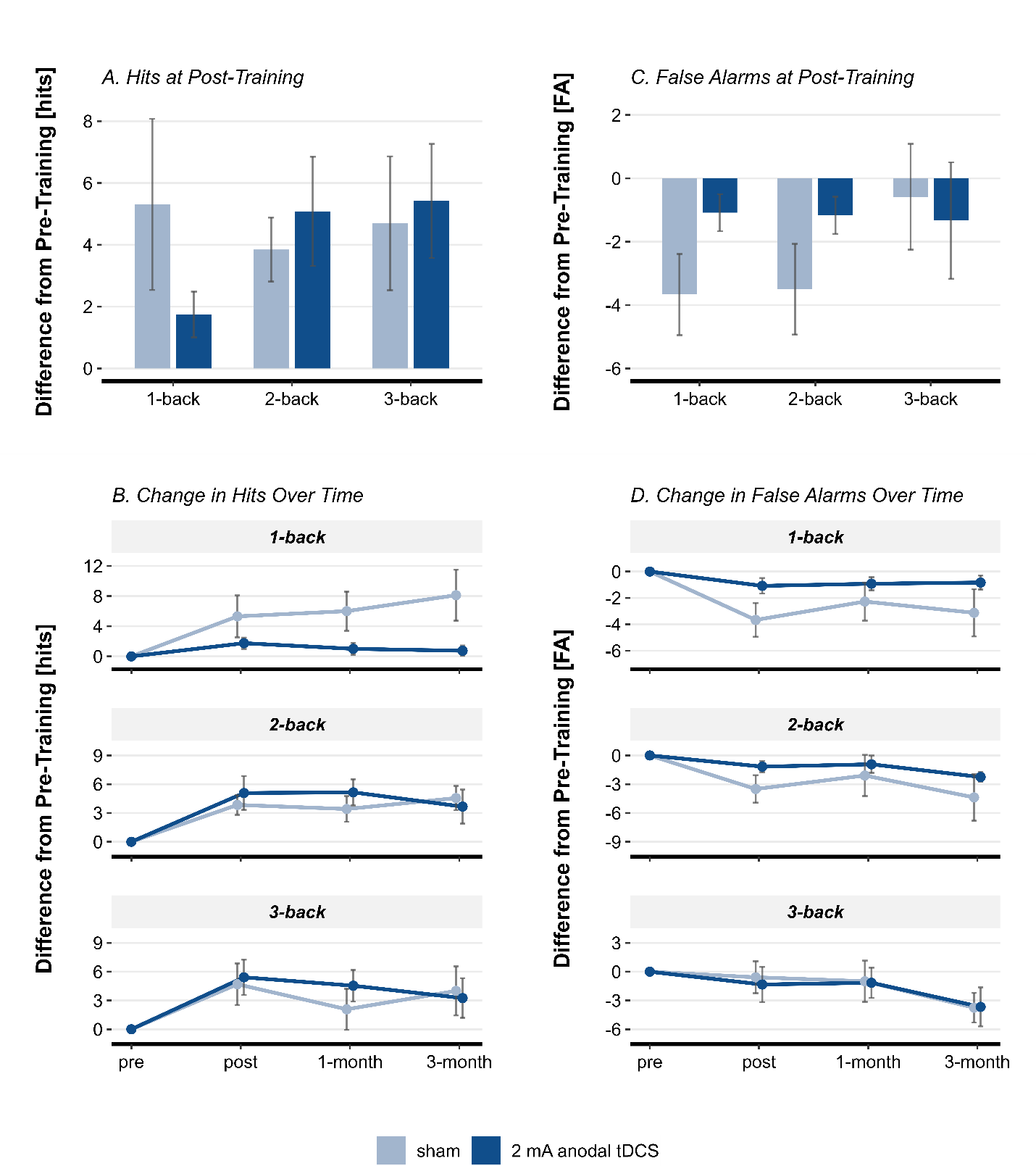 |
| --- |
| **Figure S1.** Group Differences in Hits and False Alarms Following Adaptive N-Back Training with tDCS. **A.** Mean difference in hits from pre-training to post-training, split by condition and n-back level. **B.** Mean hit difference from pre-training across all sessions, split by condition and n-back level. **C.** Mean difference in false alarms from pre-training to post-training, split by condition and n-back level. **D.** Mean hit difference from pre-training across all sessions, split by condition and n-back level. Error bars represent standard error. |

| 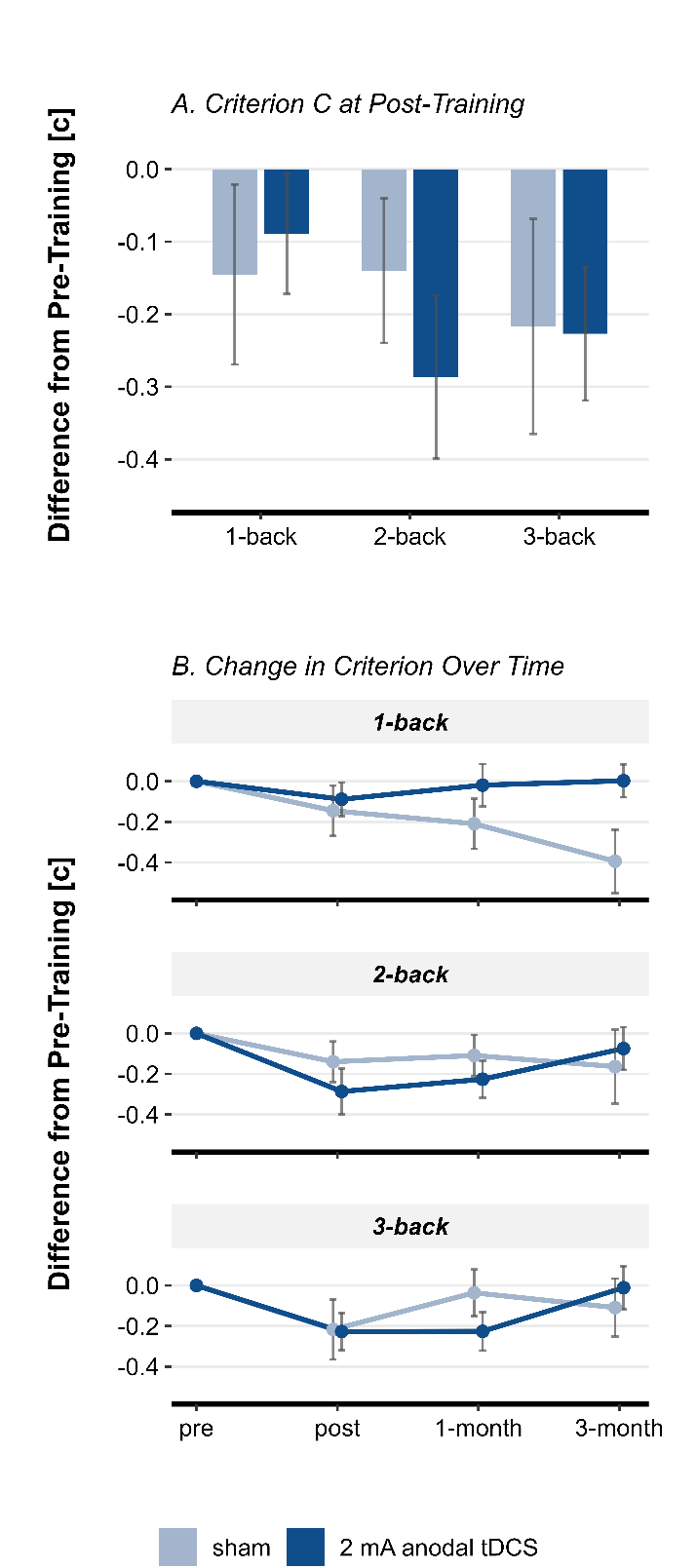 |
| --- |
| **Figure S2.** Group Differences in Criterion C Following Adaptive N-Back Training with tDCS. **A.** Mean difference in criterion c from pre-training to post-training, split by condition and n-back level. **B.** Mean criterion difference from pre-training across all sessions, split by condition and n-back level. Error bars represent standard error. |
| 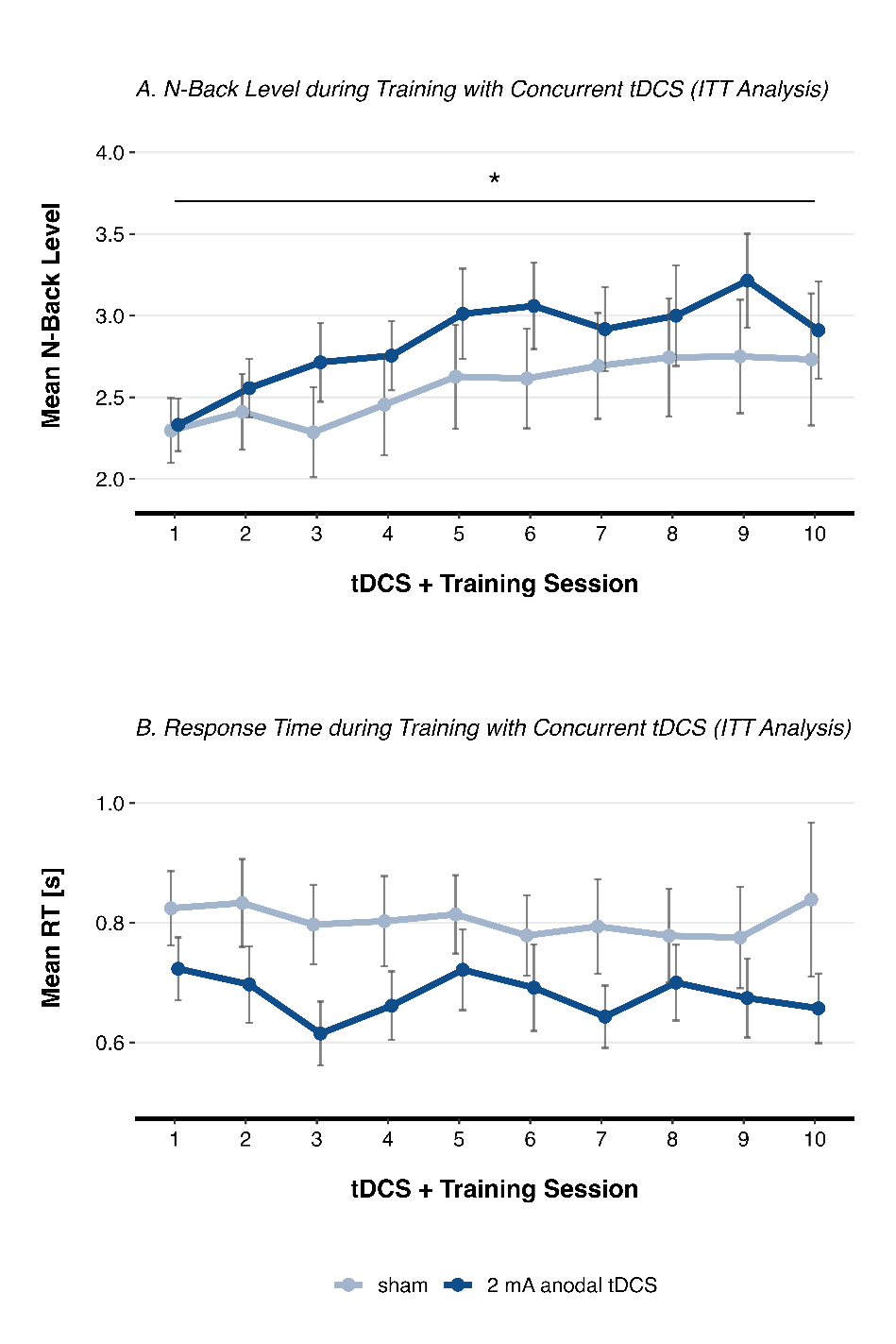 |
| **Figure S3.** IIT Analysis. Group Differences in Adaptive Spatial N-Back Training with Concurrent Stimulation. **A.** Group means of participant-level mean n-back level during each training day. The p-value represents the interaction effect of condition and session. Error bars represent standard errors. **B.** Group averages of participant-level mean response times during each training day. The p-value represents the main effect of condition. Error bars represent standard errors.  * p < 0.050 |
| 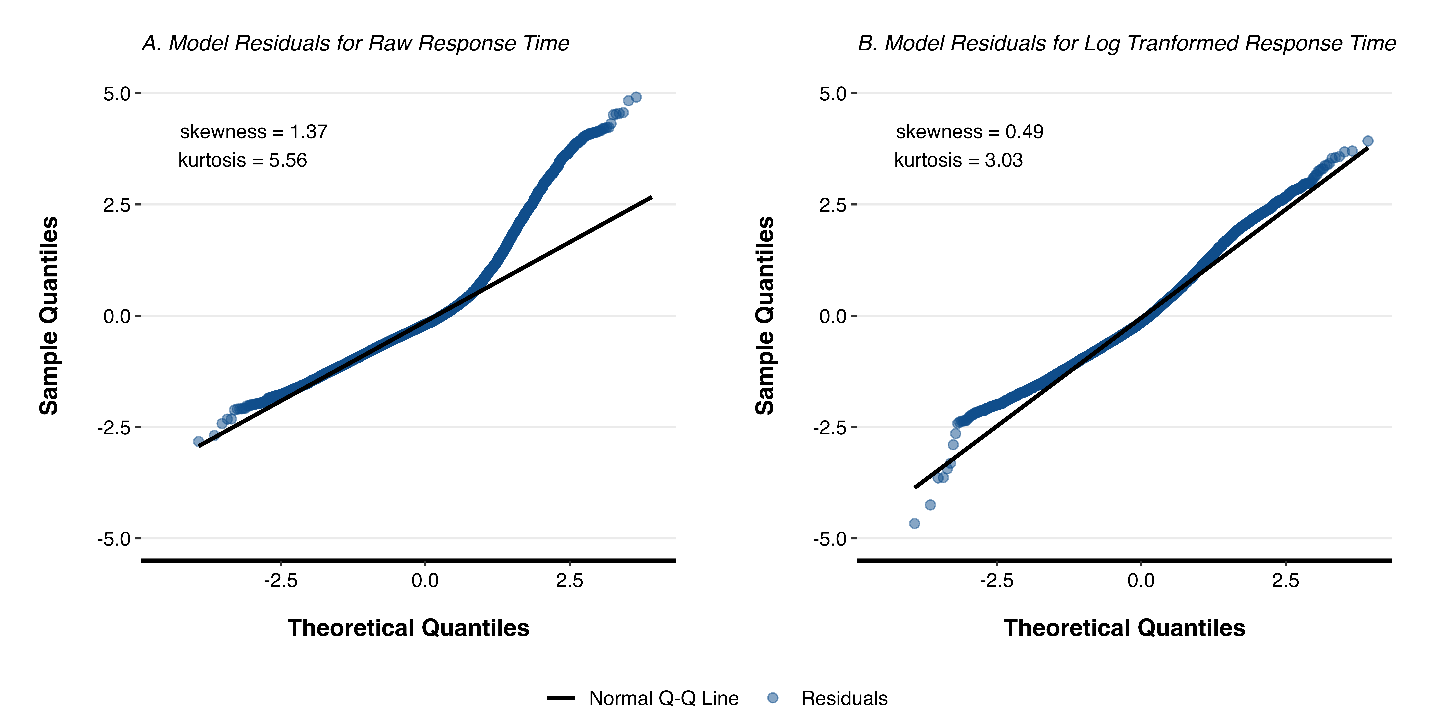 |
| **Figure S4.** Quantile-Quantile (Q-Q) Plots of Linear Mixed Model Residuals for Response Time during Training. **A.** Q-Q plot for the initial model on raw response time data demonstrating a deviation from the normal distribution. **B.** Q-Q plot of log transformed response time data, demonstrating limited deviation from the normal distribution. |
