# Supplementary material for: Investigating the effects of transcranial direct current stimulation (tDCS) on working memory training in individuals with schizophrenia
Source: Schizophrenia (Heidelb). 2025 Jul 24;11(1):106. doi: 10.1038/s41537-025-00647-5 (PMC12290004; doi:10.1038/s41537-025-00647-5)
Supplement: Supplementary file 2 — CONSORT Flowchart [file 41537_2025_647_MOESM2_ESM.docx]

**Investigating the Effects of Transcranial Direct Current Stimulation (tDCS) on Working Memory Training in Individuals with Schizophrenia**

**CONSORT 2010 Flow Diagram**

PP Analysis (n=13)
♦ Excluded from analysis (n=1)
(post assessment missed)

ITT Analysis (n=14)

PP Analysis (n=13)
♦ Excluded from analysis (n=1)
(post assessment missed)

ITT Analysis (n=14)

Excluded (n=385)

♦  Not meeting inclusion criteria (n=354)

♦  Declined to participate (n=31)

Allocated to intervention (n=18)

♦ Received allocated intervention (n=14)

♦ Did not receive allocated intervention (n=4)

- Cognitive Task was not correctly administered

Allocated to intervention (n=19)

♦ Received allocated intervention (n=14)

♦ Did not receive allocated intervention (n=5)

- Cognitive Task was not correctly administered

## Follow-Up

## Analysis

Lost to follow-up (n=1)

- Post assessment missed

Lost to follow-up (n=1)

- Post assessment missed

## Enrollment

## Allocation

Randomized (n= 37)

Assessed for eligibility (n=422)
